# Aging Diminishes Mucociliary Clearance of the Lung

**DOI:** 10.20900/agmr20220005

**Published:** 2022-06-27

**Authors:** Kristina L. Bailey

**Affiliations:** 1 Division of Pulmonary, Critical Care and Sleep Medicine, Department of Internal Medicine, University of Nebraska Medical Center, Omaha, NE 68198-5910, USA; 2 Research Service, VA Nebraska-Western Iowa Health Care System, Omaha, NE 68105, USA

**Keywords:** elderly, pneumonia, bronchiectasis, COPD, senescence, cilia, mucus

## Abstract

Healthy aging leads to a decrease in mucociliary clearance of the lung. Mucociliary clearance is an essential innate immune defense to protect against inhaled particles and microbes. Mucociliary clearance can be affected by changes in cilia function as well as mucus quantity and qualities. With aging, cilia beat frequency slows and there are changes to the characteristics of mucus. These decreases in mucociliary clearance may lead to lung infection such as pneumonia or airway diseases such as bronchiectasis or Chronic Obstructive Pulmonary Diseases.

## INTRODUCTION

**Figure 1.**
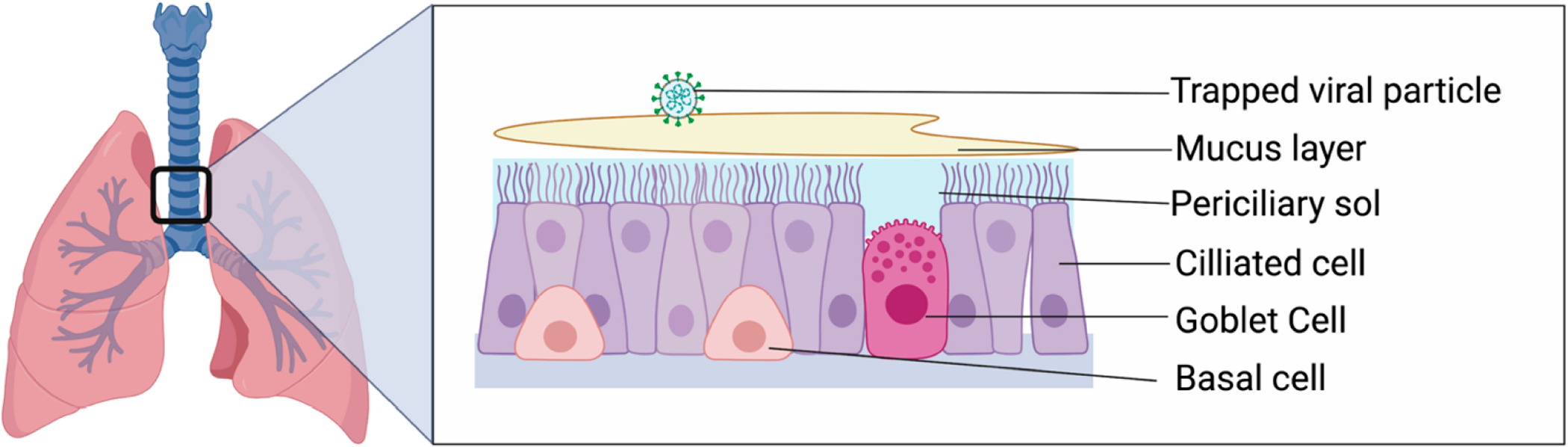
Overview of the airway epithelium. The large conducting airways of the lung are lined by airway epithelium made up of several different types of cells. This includes ciliated cells that propel mucus out of the lung, goblet cells that produce mucus, and basal cells that act as progenitor cells.

The lung is exposed to a myriad of substances with every breath we take. To protect itself from pollutants, dust, particulate matter, allergens, viruses, bacteria, and fungi that exist in the air around us, the lung has evolved a highly tuned innate immune system. One of the first lines of defense against inhaled matter is mucociliary clearance, which is performed by the airway epithelium of the trachea and the central conducting airways (Figure 1).

## NORMAL MUCOCILIARY CLEARANCE

The conducting airways of the lung are lined by ciliated airway epithelial cells. The ciliated cells are covered by a thin periciliary sol layer that is approximately the same height as the cilia (7 μm) [1]. The periciliary layer is low viscosity and facilitates ciliary beating [2]. Interspersed with the ciliated cells are mucus-producing cells. In the conducting airways, goblet cells are the most prevalent mucus-producing cell. The apical cytoplasm of goblet cells is filled with membrane-bound secretory granules filled with mucins. These granules are secreted to form the mucus layer. Mucus is a thick, gel-like material that consists of water, salts, mucins, proteoglycans, lipids, and proteins [3]. This blanket of mucus is free-floating over the respiratory epithelium. When an insoluble foreign substance is inhaled and deposits in the airway, it is trapped in a blanket of mucus. The cilia then beat in a coordinated manner to expel mucus from the lungs. The cilia tips touch the mucus layer on the forward stroke, then bend slightly on the reverse stroke, so that the cilia pass beneath the mucus layer [4]. Cilia beat at 12–15 Hz in the healthy human lung, leading to a forward/upward motion of 4–20 mm/min [5,6].

Cough is also an important mechanism of clearing the airways. In humans, cough increases as mucociliary clearance slows [7]. Impairment in cough sensitivity can lead to recurrent pneumonia [8].

Mucociliary clearance can be affected by changes in the quantity, viscosity, or composition of mucus or changes in ciliary number, structure, beating or coordination. Aging can cause changes in many of the mucociliary clearance apparatus components, leading to a higher propensity for chronic lung disease and infection with aging.

## MUCOCILIARY TRANSPORT RATES IN HEALTHY AGING

Mucociliary transport rates are known to decline with aging [9]. Multiple studies have shown slowed mucociliary transit times in older people in both the upper and lower airways. Lower airway tracheal mucus velocity can be measured by depositing radiopaque Teflon discs into the lower airways and following their motion using fluoroscopy. In younger subjects, the discs move at 10.1 ± 3.5 mm/min. In older subjects, the movement is roughly half as fast, at 5.8 ± 2.6 mm/min [10]. Likewise, tracheal mucus velocity can also be measured by injecting radiolabeled human albumin into the trachea and then recording the motion of the bolus using a gamma camera. Using this technique, younger volunteers (<50 years) had a tracheal mucus velocity of 10.7 mm/min, while older volunteers (>50 years) had a slower tracheal mucus velocity of 6.5 mm/min [11]. Small airways clearance has also been measured by using slow inhalation of radiolabeled 6 μM Teflon particles in non-smoking subjects aged 20–80. In these studies, older subjects retained more Teflon particles at 1 h and up to 21 days later [12]. Older (13–16 years) beagle dogs also have slower tracheal mucociliary transport times (2.9mm/min) than younger (2–3 years), dogs (9.7 mm/min) [13].

Upper airway mucociliary clearance can also be measured non-invasively by measuring nasal saccharine transit times (NSTT). NSTT has been shown to correlate with tracheobronchial transit times [14]. NSTT is measured by placing a small amount of saccharine in the nose, then timing how long it takes for the subject to taste the saccharine in the posterior oropharynx [15]. De Oliveira-Maul et al. demonstrated a 2% increase in NSTT with each year of age [16] in patients recruited from an Internal Medicine Clinic. In this cohort, 50% of those over 60 had prolonged NSTT (>12 min) compared to 23% of those younger than 40. Likewise, they saw slower transit times in patients with diseases associated with aging, such as hypertension and diabetes [16]. In other studies, NSTT nearly triples between age 20 and 80, from 7 min to over 20 min [17]. This is true for both males and females [18].

As women age, there are dramatic shifts in hormones in both estrogen and progesterone during perimenopause. In a study comparing premenopausal (mean age 39) and postmenopausal women (mean age 49), they measured a significant difference in clearance time using nasal saccharine transit time [19]. While the premenopausal women, on average, had nasal saccharine transit times at 11.4 min, while postmenopausal women had prolonged nasal transit times of 16.7 min [19]. It is not clear if these changes are due to aging alone, or if hormonal changes are responsible. It is likely that both changes contribute. There is some data that hormonal changes can change cilia beating. In particular, progesterone exposure in vitro in human cells derived from females is known to slow ciliary beat frequency [20]. In mice, estrogen exposure has also increased cilia beating through estrogen receptor alpha-36 [21]. Increases in the expression of the progesterone 4 receptor is known to slow cilia beating and may be the mechanism of the slowing that occurs after menopause [22].

A myriad of changes in the airway epithelium can contribute to the slowing of mucociliary transport we measure in aging. There are structural changes to the airways at the cellular level and at the level of individual cilia. There are decreases in ciliary motion (Cilia beat frequency), as well as changes in mucus composition that contribute to the changes in mucociliary transit times. We will examine each of these in turn.

## DECREASES IN THE NUMBER OF CILIATED AND BASAL CELLS IN THE AIRWAY

Single-cell transcriptomics performed in mice suggests an increase in the number of ciliated cells in the airway epithelium of aged mice [23]. However, careful phenotyping of murine airways reveals a decrease in the total number of cells in the airway, accompanied by a decrease in both ciliated cells with aging [24]. A decrease in the number of ciliated cells has the potential to diminish the effectiveness of mucociliary clearance, but this has not been tested in aging.

There is also a decrease in the number of murine basal cells in the older airways [24]. In the airways, basal cells function as stem cells for the airways [25], and a decrease in basal cells could potentially diminish the ability of the airway epithelium to repair itself after damage, which could also drive changes in mucociliary clearance.

While the changes observed in the murine airway epithelium with aging are fascinating, it is not known whether the distribution of airway ciliated and basal cells changes in human aging. However, a study designed to count the number of basal cells in normal airway epithelium did not report systemic differences based on age, but that was not the focus of the study [26].

## ULTRASTRUCTURAL CHANGES TO THE CILIA WITH AGING

**Figure 2.**
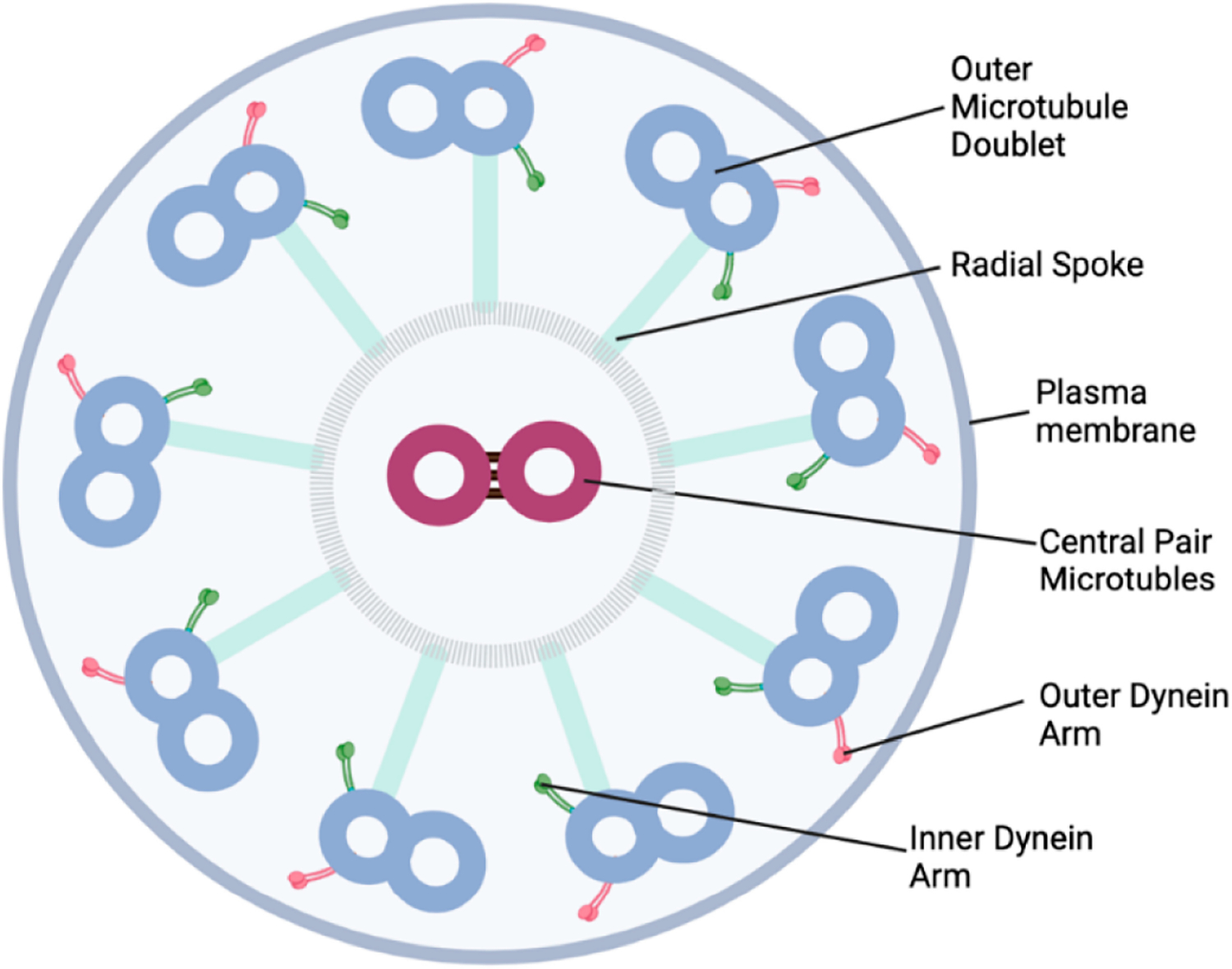
Normal structure of a ciliary axoneme. This structure is typical of the middle zones of the axoneme from motile cilia. It is often referred to as a 9 + 2 structure. This refers to the 9 outer tubule doublets and 2 central microtubules.

Aging leads to an increased frequency of ultrastructural changes in the cilia. A normal cilium has a cross-sectional ultrastructure containing two central microtubules and nine peripheral microtubule doublets (Figure 2). The ultrastructure of cilia can be analyzed using transmission electron microscopy (TEM). When TEMs from younger (11–39 years) and older donors (>40 years) were compared, 2.5% of older donors had the ultrastructural change of a single tubule, instead of the doublet tubules shown in Figure 2, vs 0.8% in younger donors [17]. These acquired ultrastructural changes can potentially alter ciliary function [27]. However, in this study, the ultrastructural changes did not correlate with ciliary beat frequency [17].

## CILIARY MOTION

There is ample evidence that cilia beat frequency (CBF) slows with aging in several species, including humans [28], mice [29–31] and guinea pigs [32]. Cilia from human bronchoscopic biopsies of the lower airways slow with age from 14.6 Hz in subjects younger than 35 to 12.9 Hz in those over the age of 65 [28]. Larger studies have measured CBF from ciliated cells harvested from the nose. Tracheal and nasal CBF have been shown to closely correlate to each other [33]. CBF from human nasal epithelial cells also slow as a function of age. In this study, older subjects had significantly slower nasal CBF, with an approximately 3 Hz decline between those in their 20’s to those over the age of 65 years [17]. The phenotype of CBF slowing with aging is maintained in airway epithelial cells cultured at air-liquid interface, with a decrease in CBF from 7.01 Hz in younger cells to 4.69 in older cells [30]. The cilia slowing was due to increased oxidative stress in the older cells, which activated Protein Kinase C epsilon (PKCε) [30]. The CBF in these aged cells was found to speed up when PKCε was inhibited chemically or using siRNA [30]. This mechanism was also found to be important in murine aging [34].

## CHANGES IN MUCUS WITH AGING

In addition to changes in cilia themselves, aging can also induce changes to the composition of mucus. Very little study of changes of mucus qualities have been performed in humans. There are a few small studies that tried to address this.

Investigators collected mucus from endotracheal tubes from patients from 1–64 years old without pulmonary disease after elective surgery (*n* = 27). They measured no differences in viscoelastic properties or transport properties of the mucus with aging [35]. Another study measured mucus clearability in younger and older human subjects by measuring the number of millimeters nasal mucus was displaced in a sneeze machine. There were no differences in mucus clearability between younger and older subjects [16]. Likewise, there were no differences in mucus contact angle, a measure of surface tension of mucus, between healthy younger and older patients [16].

Mucin 5B (MUC5B) is a major mucin in both murine and human lungs. It has been reported to impair mucociliary clearance both when it is overexpressed [36] and underexpressed in mice [37,38]. MUC5B is decreased in the lungs of older mice (24 months) compared to younger mice (3 months) leading to deficiencies in mucociliary clearance [37]. Changes in MUC5B are also associated with pulmonary fibrosis in patients over the age of 50 [39].

Changes in chloride levels in the airway surface liquid layer have been measured in aging mice. Chloride secretion is an important mechanism in maintaining a hydrated airway surface for efficient mucociliary clearance. In older mice, chloride secretion is diminished [37], which could potentially lead to thickened mucus that is difficult to clear.

Krebs von den Lungen-6 (KL-6) is a high molecular weight mucus glycoprotein. It is one of the subtypes of muc1 glycoproteins. Muc 1 is present at low levels in normal airways [40]. Muc1 increases, however, with smoking [41], airway inflammation [42], and infection [40]. Plasma levels of KL-6 are increased in older human subjects (age 35–79) compared to younger subjects (age 18–22) [43]. The increase in KL-6/muc1 in aging was thought to be due to oxidant stress [43]. Interestingly, high serum levels of KL-6 are also associated with idiopathic pulmonary fibrosis, a disease associated with aging [44,45].

In summary, mucus changes in human aging have been poorly studied. Although there are some animal studies that suggest that changes may occur, further research in humans is necessary to determine how much of a role mucus changes play in the breakdown of pulmonary innate immunity in aging. Currently, the data support a stronger role for ciliated cells in humans.

## LUNG CONDITIONS ASSOCIATED WITH AGING AND DECREASED MUCOCILIARY CLEARANCE

There are a variety of lung conditions that are associated with impaired mucociliary clearance. Many of these conditions are also associated with aging. This includes pneumonia, bronchiectasis, and COPD.

### Pneumonia

Inadequate mucociliary clearance can lead to the retention of viral particles or bacteria, leading to pneumonia. Older age is a well-established risk factor for the development of pneumonia [46]. Over half of hospitalizations for pneumonia occur in those over the age of 65 [47]. In a small Japanese study, nursing home patients that had previously had pneumonia had longer mucociliary transport times than nursing home patients that had not previously had pneumonia [48], suggesting that impaired mucociliary clearance can predispose to pneumonia.

### Bronchiectasis

Bronchiectasis is a chronic, non-reversible dilation and thickening of the airways. Bronchiectasis is a known complication of primary ciliary dyskinesia, a condition where the cilia are paralyzed, and mucociliary clearance is significantly impaired. Bronchiectasis (non-cystic fibrosis) increases markedly after the age of 60–70 [49]. The highest prevalence of bronchiectasis is reported in those over the age of 75 [50]. Increasing age is also recognized as an independent risk factor for bronchiectasis severity [51]. Likewise, sirtuin 1 (SIRT1) expression, which is frequently downregulated in aging, is downregulated in peripheral blood of bronchiectasis patients as well as in their airways [52]. In addition, telomere length was shortened in bronchiectasis patients compared to controls [52].

### Chronic Obstructive Pulmonary Disease (COPD)

The most common cause of COPD is smoking. However, occupational exposures and other frequent inhaled exposures can also cause COPD [53]. COPD is also thought to be a disease of accelerated aging [54–56]. COPD is 2–3 times more prevalent in those over 65 [57]. Increased mucus production and difficulty with mucociliary clearance are common symptoms of COPD. When mucociliary clearance has been measured in COPD patients, it is shown that clearance of radioactive particles is dramatically decreased from 30% clearance in normal controls to 4% clearance in those with COPD [58,59]. These differences were thought to be related to airways clearance [60].

## CONCLUSIONS

In conclusion, mucociliary clearance decreases with aging through a variety of different mechanisms that include structural changes in the airway epithelium, changes in cilia function as well as changes in mucus quality. These changes have the potential to contribute to clinical problems such as lung infections, bronchiectasis and COPD. As the number of people over the age of 65 continues to grow, understanding the effects of aging in the lung, and its consequences is important.

## Data Availability

No data were generated from the study.

## ABBREVIATIONS

CBF: Cilia Beat Frequency
COPD: Chronic Obstructive Pulmonary Disease
Hz: Hertz
KL-6: Krebs von den Lungen-6
MUC1: Mucin 1
MUC5B: Mucin 5B
NSTT: Nasal Saccharine Transit Time
PKCε: Protein Kinase C epsilon
siRNA: Small interfering RNA
TEM: Transmission Electron Microscopy
